# Diagnosis of Primary Colorectal Carcinoma with Primary Breast Cancer: Associations or Connections?

**DOI:** 10.7759/cureus.4287

**Published:** 2019-03-21

**Authors:** Simcha Weissman, Jefferey Sebrow, Hector H Gonzalez, Michael J Weingarten, Samuel Rosenblatt, Tej I Mehta, Rishi Thaker, Michael Krzyzak, Saad Saleem

**Affiliations:** 1 Internal Medicine, Touro College of Osteopathic Medicine, New York, USA; 2 Biochemistry & Molecular Biophysics, Newyork-Presbyterian/columbia University Irving Medical Center, New York, USA; 3 Internal Medicine, Florida Atlantic University Charles E. Schmidt College of Medicine, Boca Raton, USA; 4 Pain Management, State University of New York Upstate Medical University, Syracuse , USA; 5 Internal Medicine, State University of New York Upstate Medical University, Syracuse, USA; 6 Radiology, University of South Dakota Sanford School of Medicine, Sioux Falls, USA; 7 Internal Medicine, Staten Island University Hospital, New York, USA; 8 Internal Medicine, Mercy St. Vincent Medical Center, Toledo, USA

**Keywords:** colorectal carcinoma, breast cancer, multiple primary malignant neoplasms

## Abstract

Introduction

Although once very uncommon, multiple primary malignant neoplasms (MPMN) are becoming an increasingly popular subject in medical literature. With 182,000 new diagnoses per annum, breast cancer is the most frequently diagnosed cancer amongst women in the United States. Colorectal cancer remains the second most commonly diagnosed cancer in females, and the third in males worldwide.

Methods

In order to gather literature on synchronous and metachronous occurring breast and colon cancer, we searched PubMed using keywords such as 'colorectal cancer', 'breast cancer', and 'MPMN'. We searched through case reports, case series, clinical trials, letters to the editor, and retrospective series. We included any manuscript in English published between January 1990 and January 2019. The articles featured patients who had primary colorectal cancer with primary breast cancer. Articles featuring patients with more than two malignancies or malignancies other than colorectal and breast cancer were excluded. Furthermore, any metastatic cancers were excluded as well. This narrowed our search down from over 100 manuscripts to just four.

Results

Fortunately, the prognosis was found to be no different for these patients with MPMN assuming diagnosis and treatment are performed in a timely fashion. Additionally, it appears that although a patient with one primary cancer is at a greater risk for the development of a second cancer, it is still an odd phenomenon and thus an unlikely occurrence.

Conclusion

Detection of one cancer increases the odds of detecting another cancer. Hence, it is important to consider the possibility of a synchronous tumor in a patient with a newly diagnosed colon tumor, as well as to not only consider disease recurrence when following up post-resection.

## Introduction

Colorectal carcinoma is the third-most common cancer in the world. It is the second-most common cause of cancer-related mortality after only lung cancer. Approximately 5% of colon cancer patients have an additional primary cancer. In comparison, breast cancer has a mortality rate of 14% in women and is responsible for 30% of all cancer diagnoses in women, making it the single-most common cancer for women worldwide. Approximately 3% of breast cancer patients have an additional primary cancer. When the multiple primary cancers occur within a six-month period, they are said to be synchronous, while cancers that occur outside of that window are termed metachronous. The frequency of multiple primary malignant neoplasms (MPMN) is approximately 0.7-11% [1]. In today’s age and era, diagnoses of multiple primary malignant neoplasms are becoming more frequent, although the diagnosis is still pretty rare. Thankfully, the prognosis has not proven to be worse in this subset of patients. Interestingly, the breast cancer susceptibility gene 1 (BRCA1) and breast cancer susceptibility gene 2 (BRCA2) were found to confer increased susceptibility to colon cancer in a patient with breast cancer under the age of 50 years [2]. Furthermore, the mutS homolog 6 (MSH6) and homolog 2 mismatch repair system component (PMS2) genes, apart of Lynch syndrome, increase patients' susceptibility for simultaneous diagnoses. In fact, as many as 15% of colorectal cancers are a result of genomic micro-satellite instability, a phenomenon of deoxyribonucleic acid (DNA) mismatch repair occurring primarily in Lynch syndrome [3]. Hence, these array of genes imply a strong link between these two very different cancer groups. Additionally, it is becoming increasingly apparent why we find an increase in dual carcinoma diagnoses in a single patient. Perhaps, the simple explanation is the fact that nowadays patients are living longer, and so, the likelihood of cells mutating uncontrollably increases indefinitely. Also, as shown in the literature, a large percentage of patients with breast cancer, in particular, become diagnosed with another primary cancer, possibly owing to the fact that they contain cells with intrinsic power to replicate uncontrollably [4].

## Materials and methods

In order to gather literature on synchronous and metachronous occurring breast and colon cancer, we searched PubMed using key works such as 'colorectal cancer, 'breast cancer', and 'MPMN'. We searched through case reports, case series, clinical trials, letters to the editor, and retrospective series. We reviewed these manuscripts, as well as related articles mentioned in the references. We included any manuscript in English published between January 1990 and January 2019. The articles included featured patients who had primary colorectal cancer with primary breast cancer. Articles featuring patients with more than two malignancies or malignancies other than colorectal and breast cancer were excluded. Furthermore, metastatic cancers were excluded as well. This narrowed our search down from over 100 manuscripts. We thus were left with four manuscripts that were incorporated into this systematic review.

## Results

In one case reported, a 57-year-old male was taken to the operating room for emergency surgery after a diagnosis of intestinal obstruction. During hospitalization, breast ultrasound was performed, revealing a mass lesion in the right breast. Upon histopathology from a biopsy of the left-sided colectomy, colon cancer was diagnosed [5]. Aside from the adenocarcinoma being left-sided, the more common trend in the literature was that the hospitalization for the first primary cancer allowed for a quick diagnosis of the second one. In another case, colon cancer was detected by computed tomography (CT) scan while attempting to stage a patient's breast cancer [6]. Thus, the breast cancer diagnosis can be viewed in a positive light, as the stimulus for detecting her underlying colon adenocarcinoma. In another case reported, colonoscopy was performed due to rectal pain and bleeding, and a hepatic flexure tumor was detected. Pathologic examination of the biopsy revealed adenocarcinoma. CT scan of the thorax and abdomen, performed for staging, incidentally revealed a mass on the left breast [7]. Again, we see that a patient asymptomatic for one cancer can have a mass detected while staging the other primary tumor. In another case, a 50-year-old man presented with symptoms of rectal adenocarcinoma. During a physical examination on one of his visits, a synchronous, asymptomatic cancer of the left breast was palpated and then diagnosed with imaging [8]. Once again, we see how proper physical examination and/or proper post-cancer imaging can help hasten the findings and thus improve the prognosis of the second primary tumor.

## Discussion

The diagnosis of colon cancer most often begins with a colonoscopy after a patient presents with concerning symptoms, indicating possible disease; ideally, the test should be performed as a primary prophylaxis, namely a screening. Several randomized controlled clinical trials demonstrated that an annual fecal-occult blood test can, in fact, lower the rate of colon cancer-related mortality by a staggering 16% [9]. A colonoscopy provides for the most definitive diagnosis due to its ability to both localize and excise suspicious polyps. Colonoscopies are also highly valued for their efficacy in discovering synchronous tumors. In a study by Kim et al., preoperative identification of synchronous lesions altered the planned surgery in 14% of the patients studied. Moreover, intra-operative colonoscopy has been proven to be useful in detecting MPMN in cases where a preoperative colonoscopy is not feasible [10]. Less often, endoscopies and/or barium enemas are diagnostic as well. 

The American Joint Committee on Cancer (AJCC) and the International Union for Cancer Control (UICC) uphold the tumor-nodes-metastasis (TMN) classification system as the standardized rubric in staging cancer. The TMN system comprises five stages, the latter four of which are divided into two or three sub-stages and are generalized as follows: stage 0, the cancer is localized to the mucosal level; stage I, the cancer has penetrated as deep as the muscularis propria but has not spread to nearby organs or lymph nodes; stage II, penetration of colon and rectal wall and/or invasion of up to three nearby lymph nodes or the adjacent regions of adipose tissue; stage III, the pathological characteristics of stage II and additional invasion of up to six nearby lymph nodes or its adjacent fatty tissue and/or intrusion into nearby organs; stage IV, metastatic invasion of at least one distant organ, distant lymph node, or distant regions of the peritoneum.

Diagnosing breast cancer begins with a thorough examination of the breasts and axillary lymph nodes. Subsequently, the patient will undergo a mammogram. If the mammogram presents suspicious findings, a biopsy will then ensue by removing a lump in partial (core biopsy or vacuum-assisted biopsy) or completion (excisional biopsy). Alternatively, a fine needle aspiration biopsy may be performed in which fluid or a minute amount of tissue is removed from the potentially cancerous tissue. A pathologic analysis will then follow, which will allow for the determination of cancerous and genetic characteristics. Both magnetic resonance imaging (MRI) and ultrasound imaging studies are used in addition to mammography to stage and evaluate the progression of breast cancer. According to an article by McDonald et al., modern medicine has significantly transformed in the era of personalized medicine. Due to this paradigm shift, we no longer follow a one-size-fits-all approach. Now, sophisticated diagnostics, such as molecular imaging and genomic expression profiles, enable improved tumor characterization [11]. Hence, in this age and era, the diagnosis of synchronous and metachronous lesions are only becoming more and more prevalent. 

As with other cancers, the staging of breast cancer is based on the five stages of the TNM classification system. These stages are described as follows: stage 0, also referred to as non-invasive cancer, where the cancer is strictly localized to the glands or ducts of the breast tissue without any indication of cancer cells in nearby organs or lymph nodes; stage I, an invasive tumor of up to 2 cm, whereby the cancer has spread beyond the glands (invasive glandular carcinoma) or ducts (invasive ductal carcinoma) but is localized to surrounding breast tissue, or when microscopic clusters of cancer cells between 0.2 and 2 mm are present within the axillary lymph nodes; stage II, the presence of a breast tumor between 2 and 5 centimeters and/or the significant occurrence of disease in up to three axillary lymph nodes; stage III, a breast tumor of varying size with the cancer having spread to a minimum of four and maximum of nine axillary lymph nodes; stage IV, the cancer having metastasized to other organs or distant lymph nodes.

The treatment for colorectal cancer depends largely on the stage of the cancer. For cancers in the early stages (stages 0, I, II), surgical interventions to remove the tumor and any affected lymph nodes are usually adequate for successful treatment and subsequent remission. Under certain circumstances, in situations where the cancer is suspected to have an especially high risk of recurring, stage II colorectal cancer patients may also receive chemotherapy. No universally accepted rule exists, however, regarding chemotherapy for stage II colorectal patients. For stage III colorectal cancers, whereby the cancer has spread to lymph nodes, the standard treatment includes surgical removal of the tumor, which often includes sections of the surrounding colon or rectum, followed by chemotherapy. The chemotherapeutic regimens that have been shown to be particularly effective at treating later stage metastatic colorectal cancers include: fluorouracil, leucovorin, oxaliplatin (FOLFOX), or fluorouracil, oxaliplatin irinotecan, leucovorin (FOLFOXIRI); or fluorouracil, leucovorin, and irinotecan (FOLFIRI). More recently, capecitabine and oxaliplatin (CapeOx) have been shown to be effective in certain situations. There are a number of important factors that guide the chemotherapeutic approach for patients with metastatic colorectal cancer. For unfit patients and those with extensive comorbidities, supportive care alone is recommended if they are unable to tolerate systemic chemotherapy. If they are able to tolerate systemic chemotherapy, the regimen includes fluoropyrimidine with or without bevacizumab. Contraindications to bevacizumab include: active bleeding, recent arterial thromboembolic events (within the last year), or a major surgery within the last month. For fit patients, the major considerations that determine the preferred chemotherapeutic regimen are: rat sarcoma (RAS) and murine sarcoma viral oncogene homolog (BRAF) V600E mutation status, left or right-sided primary tumor (i.e., before or after splenic flexure), whether they are candidates for bevacizumab, and whether they can tolerate intensive therapy. For those who can tolerate intensive therapy, if they have mutated RAS or BRAF V600E, or if the primary tumor is right-sided (even if their genotype is wild-type for RAS and BRAF), then FOLFOX or FOLFOXIRI are the recommended regimens, with or without the addition of bevacizumab (depending on the contraindications listed above). For those patients with tumors wild-type for RAS and BRAF and left-sided primary tumors, FOLFOX or FOLFIRI is recommended, together with cetuximab (which is ineffective against RAS and BRAF mutations but otherwise a beneficial addition). There are slight modifications in the chemotherapeutic regimens for patients unable to tolerate such intensive therapy. For those with tumors wild-type for RAS and BRAF or left-sided primary tumors, there are a number of options for first-line treatment. These include: FOLFOX or FOLFIRI with or without cetuximab, CapeOx with bevacizumab (if the latter is not contraindicated), or fluoropyrimidine [12]. And finally, for those with mutated RAS or BRAF genes or right-sided wild-type, CapeOx with bevacizumab, FOLFOX or FOLFIRI or CapeOx without bevacizumab, or fluoropyrimidine alone is recommended.

The treatment of breast cancer is highly patient specific and strategies are still evolving. The chosen regiment is generally predicated upon numerous factors including gender, age, tumor size and grade, and the expression or absence of various endocrine and growth factor receptors in the tumor [13]. Notwithstanding this inherent variability, a number of broad treatment approaches can be outlined. Early stage breast cancer patients are advised to proceed with either breast-conserving therapy (lumpectomy) or mastectomy. Post-surgical radiation accompanies breast-conserving therapy and is indicated after mastectomy for those with a high risk of local recurrence. Once the tumor is excised and its characteristics become known, decisions about adjuvant therapy are then made. Patients with hormone-receptor positive breast cancer are encouraged to undergo endocrine therapy, with a selective estrogen receptor modulator (tamoxifen) being administered to those premenopausal, and an aromatase inhibitor being given to those postmenopausal. Alternatively, if the breast cancer is found to be human epidermal growth factor receptor-2 positive, the patient is administered trastuzumab for one year. For patients with locally advanced breast cancer, neoadjuvant systemic therapy is indicated pre-operatively, with the goal of shrinking the tumor and maximizing breast conservation.

With 182,000 new diagnoses per annum, breast cancer is the most frequently diagnosed cancer amongst women in the United States. In the female population, breast cancer makes up a disproportionally large percentage - 26% of all cancer cases - and is responsible for 40,000 deaths per year nationally [14]. According to the American Cancer Society, one in eight women will develop breast cancer in their lifetime. Breast cancer incidence rates increase with age. The most dramatic increases occur before age 50 years, during the premenopausal years. It is during this time period that incidence rates increase eight to nine percent per year, and begin to sharply decrease once menopause begins, to about two and three percent [15-16]. At or around age 70, rates begin to slightly decrease. Eighty percent of female breast cancers are diagnosed in women aged 50 years and above and fifty percent are diagnosed between ages 50 and 69 [17]. Breast cancer incidence and mortality rates show significant differences amongst different races and ethnicities. Non-Hispanic whites and non-Hispanic blacks have higher incidence and mortality rates than women of other races/ethnicities with Asian/Pacific Islander women showing the lowest rates. Though black and white women have similar rates of incidence, black women have a 42% greater mortality rate [18]. Intrinsic risk factors for breast cancer include age and race along with various genetic factors and sex hormone concentrations. The mutations BRCA1 and BRCA2 are the most notable mutations implicated in breast cancer. High endogenous estrogen levels are also well-established risk factors for breast cancer. Extrinsic risk factors include obesity and oral hormonal menopause therapy [17].

Although its prevalence continues to slowly decline in the elderly population, colorectal cancer remains the second most commonly diagnosed cancer in females, and the third in males worldwide. Interestingly, for peoples under the age of 40, there has been an increased prevalence of 2-3% over the last several years. Its incidence varies widely, with the highest rates of occurrence in North America, Australia, Europe, and New Zealand and the lowest in South Central Asia and Africa [19]. These geographical differences are thought to be predicated upon varying nutritional and environmental exposure. Additional risk factors for the disease include low socioeconomic status, increased age, and low rates of screening [20].

Colon cancer prognosis mainly depends on the staging of the carcinoma at initial presentation, the extent of lymph node involvement, histological grading of the differentiation, preoperative levels of serum carcinoembryonic antigen, the presence of distal metastasis, as well as genetic factors. Of those genetic factors, microsatellite instability, RAS, and BRAF mutations are of great importance. Five-year survival rates for colon cancer patients with stage I, IIA, IIB, IIIA, IIIB, IIIC, and IV disease between 1991 and 2000 were 93, 85, 72, 83, 64, 44 and 8 percent, respectively [21].

As with any other carcinoma prognosis, staging (which includes tumor size, lymph node involvement, and metastasis) is the most important prognostic factor for breast carcinoma. The annual risk of breast cancer recurrence was highest during the first five years (10.4%) with a peak between first and second year (15.2%) in a study over 4,000 women [22]. Patients with triple negative-estrogen hormonal receptors, progesterone hormonal receptors, and human epidermal growth factor receptor 2 carried a poorer prognosis. BRCA-mutated breast cancer is also associated with a bad prognosis.

## Conclusions

In conclusion, as illustrated in this manuscript, detection of one cancer increases the odds of detecting another cancer due to more extensive and careful physical examinations, imaging studies, and post-disease monitoring. As such, it is important to consider the possibility of a synchronous tumor in a patient with a newly diagnosed colon tumor. Thus, upon diagnosing any tumor, a meticulous physical examination should be encouraged. Additionally, it is important to not only consider disease recurrence when following up post-resection, as metachronous tumors should be amongst the differential diagnosis of a clinician. Furthermore, we very often see that periodic screening in an attempt to prevent disease recurrence may actually hasten the diagnosis of a new primary tumor.
